# Impact of Different Combinations of Processing Steps on Product Quality and Proximate Composition of *Bêche-de-mer*: A Case Study from Sri Lanka

**DOI:** 10.1155/2022/7877050

**Published:** 2022-08-09

**Authors:** G. Nishanthan, P. A. D. A. Kumara, S. B. Navarathne, D. C. T. Dissanayake

**Affiliations:** ^1^Department of Zoology, University of Sri Jayewardenepura, Gangodawila, Nugegoda, Sri Lanka; ^2^Inland Aquatic Resources and Aquaculture Division, National Aquatic Resources Research and Development Agency (NARA), Crow Island, Colombo 15, Sri Lanka; ^3^Department of Food Science and Technology, University of Sri Jayewardenepura, Gangodawila, Nugegoda, Sri Lanka

## Abstract

*Bêche-de-mer* (dried sea cucumbers) is considered as one of the most luxurious seafood in the world. The market demand for *bêche-de-mer* mainly depends on species and product quality. This study is aimed at assessing the effect of different processing methods on the product quality and nutritional composition of *bêche-de-mer*. Three sea cucumber species, *Stichopus naso*, *Holothuria spinifera*, and *Bohadschia vitiensis*, dominant in the commercial catches of Sri Lanka, were processed by following widely practiced local processing methods where 24, 48, and 24 different processing combinations were tested for each species, respectively*. Bêche-de-mer* satisfying the export quality were selected by considering their appearance and overall ranking made by exporters (9 individuals per each combination). The proximate composition of these products was assessed using standard methods. According to the exporters' prioritization, very few processing methods used in this study resulted in the export-quality *bêche-de-mer*. Both product quality and proximate composition showed considerable variations with respect to the processing methods. It is proved that even a very small change in one processing step could lead to produce poor-quality products. Processing methods are species specific, and it is important to select the most appropriate method for each species to produce high-quality *bêche-de-mer*.

## 1. Introduction

Sea cucumbers belonging to the class Holothuroidea of the phylum Echinodermata are soft-bodied, worm-like marine benthic invertebrates found at all latitudes and all depths [1, 2]. The exploitation history of sea cucumbers dates to several centuries, and sea cucumbers first became commodities in China and India more than 1000 years ago [3, 4]. As there was a lucrative market for sea cucumber products throughout the world, fishing activities were rapidly expanded into new fishing grounds and currently more than 70 countries are engaged in sea cucumber fishing worldwide, targeting around 66 species [5–7].

Sea cucumbers are served as a good source of food, especially to the Southeast Asians, mainly as *bêche-de-mer* (dried sea cucumbers), although fresh, salted, cooked, and frozen products are also available [7–13]. *Bêche-de-mer* is mainly produced by a process of cleaning, evisceration, first boiling (cooking), salting, second boiling, and drying. However, slight differences in these major processing steps were evident depending on the species and geographical regions [1, 8, 12, 14].

The sea cucumber fishery has been providing an important means of livelihood for the coastal fishing communities in Sri Lanka for centuries. Although around 21 sea cucumber species are commercially exploited in the coastal waters of Sri Lanka, there is no tradition of consuming them locally and the entire harvest is exported to Singapore, Taiwan, and China as *bêche-de-mer* [15]. In Sri Lanka, sea cucumbers are processed into *bêche-de-mer* by both domestic and industrial level processors, mainly following the major processing steps of grading, evisceration, first boiling, salting, second boiling, and drying [12] but with their own modifications to one or many steps according to their traditional knowledge and hands-on experience. Most of these modifications have been passed from generation to generation and remain undisclosed, while others are ad hoc practices done by domestic level processors.

It is well documented that the market demand for *bêche-de-mer* fluctuates with species and product quality. Appearance, color, odor, and moisture content are the key determinants of *bêche-de-mer* product quality, and these parameters are mainly influenced by processing methods [12]. According to Wen et al. [16], the nutritional composition of *bêche-de-mer* is significantly affected by the way they are processed. Although many attempts have been made to study sea cucumber processing practices, their advantages, and drawbacks [6, 12, 14, 17], only a few studies have focused on assessing the impact of different processing practices on the product quality and nutritional composition [1, 18]. Thus, this study is aimed at filling this research gap by providing a detailed analysis on how different combinations of processing steps affect the product quality and nutritional composition of three sea cucumber species, i.e., *Stichopus naso*, *Holothuria spinifera*, and *Bohadschia vitiensis*, dominating the commercial catches in Sri Lanka.

## 2. Materials and Methods

### 2.1. Sample Collection

**Figure 1:**
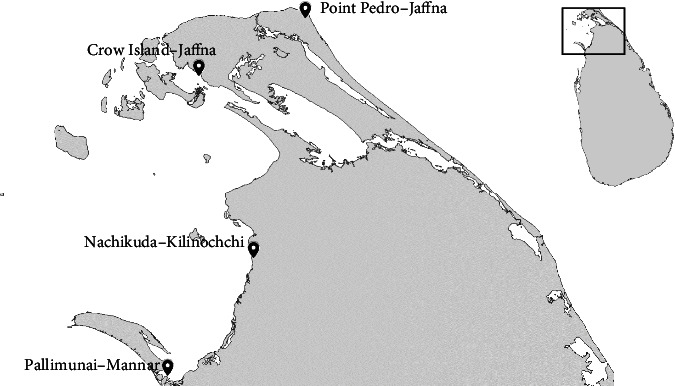
Map showing the sampling sites on the North and Northwest coast of Sri Lanka.

Fresh samples of *S. naso* (*n* = 360), *H. spinifera* (*n* = 720), and *B. vitiensis* (*n* = 360) were collected from major sea cucumber landing sites in the north and northwest coasts of Sri Lanka (Figure 1). All collected sea cucumbers were properly washed using clean seawater to remove slime and sand on the body surface and transported to the processing plants located in Colombo (transportation time = 06 − 07 hours) and Jaffna (transportation time = 01 − 02 hours), Sri Lanka under chilled conditions. As *S. naso* autolyzes its body tissue immediately after taking it out of the water, they were transported to the processing plants in seawater.

### 2.2. Experimental Design for Processing

**Figure 2:**
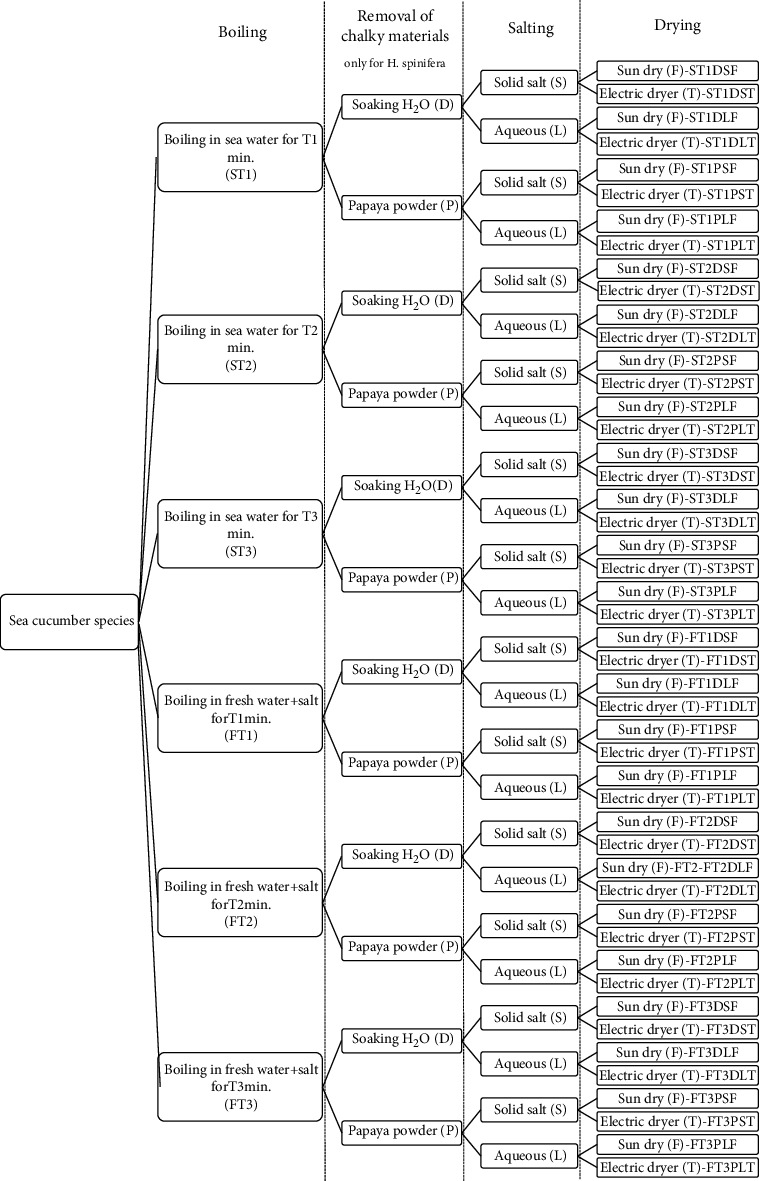
Experimental design used to process three sea cucumber species *H. spinifera*, *B. vitiensis*, and *S. naso* into *bêche-de-mer*.

The individual weight of fresh sea cucumbers was measured at the processing plants, and they were processed into *bêche-de-mer* following different combinations of processing steps used by local processors for each species. Accordingly, forty-eight alternative methods were used to process *H. spinifera* and 24 processing methods were applied to *B. vitiensis* and *S. naso* samples (Table 1 and Figure 2).

### 2.3. Effect of Processing on Product Quality

The quality of the processed samples was initially assessed based on their appearance, mainly considering the percentages of damaged, deformed (with bent and misshaped), patched, and oversalted individuals. Then, these products were packed in polythene bags according to the assigned code number and anonymously presented to exporters (*n* = 9). They were asked to select export-quality products and line them up from the highest to the least quality, respectively, by assigning numbers in ascending order starting from 1. Factors considered to select export-quality products and rank them were reported by interviewing exporters. The results were recorded separately for each species using the code number assigned to each product (Figure 2).

By considering the initial assessment and selection made by exporters, the export-quality *bêche-de-mer* were selected and ranked by averaging the individual ranking given by exporters to understand the effect of different processing procedures on the final product quality and to select the most appropriate processing practices for each species.

### 2.4. Effect of Processing on Proximate Composition

To assess the effect of different processing practices on the nutritional composition of *bêche-de-mer*, the proximate composition of the processed products was analyzed. Due to practical limitations, only the selected export-quality *bêche-de-mer* from each species were considered for this analysis. Representative samples (*n* = 5) from each selected product were taken and ground using a grinder to obtain a homogenous sample. Each sample was analyzed for percentage moisture and dry matter percentages of crude ash, crude fat, and crude protein. The moisture content was determined by drying the samples in a thermostat oven at 100 ± 5°C until a constant weight was obtained [19]. Crude ash content was determined by incinerating the samples in a muffle furnace at 550°C for 24 h [19]. The micro-Kjeldahl method with acid digestion was used to determine the crude protein content, and conversion factor 6.25 was used to convert total nitrogen to crude protein [10]. Bligh and Dyer's method was used to determine the crude fat content [20]. Each experiment was carried out in triplicate.

### 2.5. Statistical Analysis

The mean (±SD) weight of the fresh and processed samples, percentage weight loss during processing, percentages of damaged, deformed, patched, and oversalted individuals, and the exporters' ranking of each product were calculated using the MS Excel software. All proximate components, except moisture, were determined on a dry weight basis. Variations in the proximate composition of the selected products of each species were compared using analysis of variance (ANOVA) followed by Tukey's multiple comparison test. Differences were considered significant when *p* < 0.05. The statistical tests were performed with Minitab 18 for the Windows statistical package.

## 3. Results

### 3.1. Weight Changes during Processing

According to the initial assessment and overall ranking of exporters, differently processed 24 products, representing 8 products from each species, were selected as the export-quality *bêche-de-mer*. Fresh weight, processed weight, and percentage weight loss of these selected products are summarized in Table 2, and the same results obtained for *H. spinifera* (*n* = 48), *B*. *vitiensis* (*n* = 24), and *S. naso* (*n* = 24) under different processing methods are summarized in Supplementary Tables 1, 2, and 3, respectively.

The percentage weight loss of *H. spinifera* ranged from 85.89 to 94.11%, while it was from 89.45 to 93.26% and 86.54 to 93.51%, respectively, for *B. vitiensis* and *S. naso.* The results indicated that considerable weight loss of fresh sea cucumbers can occur during processing, and this mainly depends on the target species and processing practices. The processing processes like long-time boiling and drying with electric dryers have made more contribution to the weight loss of the processed products.

### 3.2. Selection and Ranking of Export-Quality *Bêche-de-mer*

The results of the initial assessment (i.e., % damaged, % deformed, % visible patches, and % salted) and the overall ranking for the processed products of *H. spinifera*, *B. vitiensis*, and *S. naso* are summarized in Supplementary Tables 1, 2, and 3, respectively.

Out of the 48 processed products of *H. spinifera*, the samples coded as F20PST, F15DLT, F10DLT, F10DST, F20PLF, F10PLF, S15DSF, and F20DSF were the selected export-quality products. Among these samples, F20DSF reported the lowest percentage of damaged (0%), deformed (0%), patched (0%), and oversalted (20%) individuals. More than half of the processing practices used to process *H. spinifera* resulted in a high percentage of damaged individuals ranging from 14 to 50%. The highest percentage of deformed individuals was reported in F15DSF (80%), followed by F20DST (71.43%, Supplementary Table 1). Seven out of the eight selected products were cooked in freshwater with added salts, and half of these products were dried in an electric dryer. Chalky materials in the majority of these products (*n* = 5) were removed by soaking in freshwater. The high percentage of salted individuals appears to be an issue in most of the processing procedures, and this is especially true when aqueous salt is used (Supplementary Table 1).

The exporters ranked the selected 8 products of *H. spinifera* as F20DSF, F20PST, F20PLF, S15DSF, F15DLT, F10PLF, F10DST, and F10DLT (Table 2; Supplementary Table 1) by considering factors such as appearance, dryness, presence of spike-like projections on the body surface, and the absence of CaCO_3_ deposits. The way of removing chalky materials and the method of drying are the main differences observed between the F20DSF and F20PST, where chalky materials in the F20DSF were removed by soaking in water and it was dried under sunlight, while papaya leaves were used to remove chalky materials in the F20PST and it was dried in an electric dryer. The results revealed that different combinations of processing steps can be used to produce high-quality *bêche-de-mer* from *H. spinifera.* Cooking in freshwater with added salts around 15-20 minutes, removing chalky materials in soaking water, salting with solid salt, and drying under sunlight for a period of 5 days can be considered as a good processing step.

The high-quality processed products of *B. vitiensis* include the samples labeled as S10ST, F10ST, F15ST, S15LT, F10LT, S20LF, S15LF, and F20LF. Of those, the S10ST sample reported 0% of damaged, deformed, patched, and oversalted individuals. When compared to *H. spinifera*, *B. vitiensis* reported a lower percentage of damaged individuals and the two lowest-quality products, F10LF and F15SF, reported only 20% of damaged individuals. However, 20 out of the 24 different processing methods resulted in deformed individuals and their percentage ranged from 0 to 85.71%. The highest percentage of deformed individuals was reported in F20ST (85.7%) followed by S10LT and S15SF (80%). The sample F15LF reported the highest percentage of visible patches (80%). It is noted that *B. vitiensis* processed using aqueous salt and/or dried under sunlight often results in dull body color than the samples processed using solid salt and electric drying (Supplementary Table 2).

By considering the brightness of the dorsal body surface, the curved nature of the ventral body surface, and the level of drying, exporters lined up the selected 8 products of *B. vitiensis* from the highest-to least-quality products as F15ST, F10ST, S10ST, S20LF, S15LF, S15LT, F10LT, and F20LF (Table 2; Supplementary Table 2). The least-quality products are characterized by dull-colored body surfaces. The results of this study revealed that *B. vitiensis* processed by boiling for 15 minutes in freshwater with added salts, salting with solid salts, and drying in an electric dryer for 15 hours generates higher-quality *bêche-de-mer* than the other processing methods.

The eight samples coded as S6LF, F6LT, F3SF, F6LF, S3LF, F6SF, F9LF, and S3SF of *S. naso* were identified as the best processed products. Two samples, F3ST and S3SF, showed 0% of damaged, deformed, patched, and oversalted individuals. The lowest quality was evident in the F3LT sample, which reported 25% of damaged and 62.5% of deformed individuals. Similar to *B. vitiensis*, *S. naso* processed using aqueous salts resulted in a dull-colored body than the samples processed using solid salts. The selected samples, except F6LT, were dried under sunlight (Table 2; Supplementary Table 3).

The exporters and processors gave their highest preference to the S3SF, followed by the F3SF (Table 2; Supplementary Table 3). The adequacy of dryness, free from deformation, and the size of the processed individuals are the primary criteria used by them to rank the processed products of *S. naso*. Similar to *B. vitiensis*, the quality products were characterized by a dull-colored external appearance.

The results revealed that different combinations of processing steps can be used to produce high-quality *bêche-de-mer* of *S. naso.* However, a 3-6-minute boiling time, salting with solid salts, and sun drying for a period of 3 days can be considered as a good combination of processing steps.

### 3.3. Proximate Composition of Export-Quality *Bêche-de-mer*

The proximate composition of the export-quality *bêche-de-mer* of *H. spinifera*, *B. vitiensis*, and *S. naso* is summarized in Table 3.

The moisture content of the selected products of *H. spinifera* ranged from 17.9% to 22.81%. The highest moisture content was reported in F10DST (22.81 ± 0.26%) and F10DLT (22.51 ± 0.05%) samples, while the lowest moisture content was evident in S15DSF (17.9 ± 0.13%). The crude protein content of the processed *H. spinifera* ranged from 55.4% to 59.21% and F10DST (59.21 ± 0.07%), F20PST (58.68 ± 0.67%), and S15DSF (58.47 ± 0.08%) reported a significantly higher crude protein content than the other products (ANOVA, *p* < 0.05). The crude ash content ranged from 30.05 ± 0.38% to 35.91 ± 0.52% reporting the highest content in F15DLT. The crude fat content of *H. spinifera* was found to be very low when compared with the other two species, and the values ranged from 1.26 ± 0.0% to 1.47 ± 0.02%. Significant variations in percentage moisture, crude protein, crude fat, and ash content were evident among differently processed samples (ANOVA, *p* < 0.05). From the nutritional point of view, the steps used to process the sample S15DSF can be considered as the most acceptable steps for processing *H. spinifera* as this product reported significantly lower moisture, higher protein, and lower fat compared to other products (ANOVA, *p* < 0.05).

The moisture content of the processed *B. vitiensis* samples was within a similar range (19.08% to 21.06%), and the highest moisture content was reported in the F10LT sample. There were no significant differences in the protein content (ranged from 49.97% to 52.56%) of the processed samples (ANOVA, *p* > 0.05). The lowest crude ash content was evident in sample F10ST (39.85 ± 0.03%, ANOVA, *p* < 0.05), and the crude fat content ranged from 1.76% to 2.04%, reporting a significantly higher fat content in F20LF (2.04 ± 0.02%) and S15LT products (2.03 ± 0.01%, ANOVA, *p* < 0.05) than the others. The sample F15ST, which received the highest ranking, has significantly lower moisture (ANOVA, *p* < 0.05), higher protein, and lower fat compared to other products.

The moisture content of the processed *S. naso* was within the range of 14.67%-17.89%, and the lowest moisture content was reported in the F3SF sample (ANOVA, *p* < 0.05). Significant differences in crude protein (48.1% to 51.9%), crude ash (42.14% to 44.62%), and crude fat (2.29% to 2.65%) contents were found among the processed samples (ANOVA, *p* < 0.05).

## 4. Discussion

The processing of sea cucumbers into *bêche-de-mer* is a largely unregulated process. However, many attempts are made continuously to study and improve the processing process of sea cucumbers as the end market value of *bêche-de-mer* is mainly dependent on product quality [17, 21].

It is reported that many countries, including the Pacific Islands [6, 14], Sri Lanka [12], and Madagascar [17], have identified the limitations of their existing processing practices. Although some attempts have been made to improve the processing quality of *bêche-de-mer* by Chong et al. [22], Duan et al. [18], and Duan et al. [1], there are still many poor-quality products in the international markets. This could be due to many reasons. As reported in this study as well as in many previous studies, processing practices vary from species to species and processors are not well aware of the most appropriate processing method for each species. Due to the lack of species-specific standard processing practices, proper awareness, and training, many different processing practices are currently used by sea cucumber processors in Sri Lanka. However, the results of this study revealed that most of these processing practices contribute to produce inferior-quality products. However, it is also proved that export-quality *bêche-de-mer* products can be produced following combinations of different processing steps.

Grading and cleaning of sea cucumbers are the first two steps used in the sea cucumber processing chain. Grading is an important activity to separate them into species as well as into different size classes. According to Purcell et al. [6], different species require different processing methods due to differences in the thickness of their body wall and the extent of water retention, and this study also supports their findings. On the other hand, larger-sized sea cucumbers require longer boiling time than smaller-sized individuals. Thus, processing without grading leads to produce poor-quality products.

Evisceration is a vital step in sea cucumber processing, and it must be carried out with great care [8, 12, 23]. Evisceration is mainly carried out to remove the internal organs, as the presence of such organs badly affects the quality of *bêche-de-mer* [14]. Evisceration is mainly done by making a small cut (2.5–4.0 cm) at the posterior end of the body. However, in some species, i.e., *S. chloronotus* evisceration is done at the ventral body surface, while in teatfish species (*H. fuscogilva* and *H. nobilis*), it is commonly done at the dorsal body surface [6, 12]. The widely practiced methods were used to eviscerate the experimental animals. The evisceration is generally carried out before the first boiling [12, 14, 22]; however, highly perishable *S. naso* was boiled before evisceration to reduce postharvest losses [22].

Boiling or cooking is the most important step in the sea cucumber processing chain as it improves the palatability, the rate of drying, and the odor of the end product [23]. According to Nishanthan et al. [12], the quality of *bêche-de-mer* mainly depends on the boiling medium and boiling time. Previous studies recommended saltwater as the preferred medium for cooking sea cucumbers [22], but this study confirms that the use of freshwater with added salt does not have any significant impact on the final product quality. However, as most of the processors tend to use seawater directly taken from adjacent coastal water bodies contaminated with domestic and industrial waste, it is good to use freshwater with added salt as the boiling medium.

The use of proper boiling time is vital for the final product's quality. The boiling time depends on several factors, such as species, size, cooking pressure, and cooking temperature [1, 24]. This study revealed that boiling time varies from species to species and longer or shorter boiling time results in overboiled or partially boiled products with a high percentage of damaged and deformed individuals. As reported in this study, these products normally receive a low market value because the shape and external appearance are some of the major factors used to rank *bêche-de-mer* [14, 18, 22, 24].

Some species, like *H. spinifera* and *H. scabra* have CaCO_3_ deposits on their bodies and these should be removed during processing [12, 17]. Different methods, including many traditional methods, are used to remove these chalky materials in many countries [6, 12, 14]. Local processors mainly use two methods to remove the chalky material of sea cucumbers. Although papaya powder is successful in removing chalky materials, as reported in Madagascar and some Pacific Island countries [6, 17], local exporters gave less preference for these products due to the lack of spike-like projections on the body surface of *H. spinifera*. The exact reason for this observation is not very clear, but it could be due to the removal of spike-like projections by the uncontrollable action of papain enzyme on CaCO_3_ deposits on the body surface [17].

Salting after the first boiling limits desiccation, minimizes weight and length losses during processing, and preserves the product by limiting microbial growth [6, 17, 22, 25]. According to Purcell [24], poorly salted *bêche-de-mer* is a regular problem for many exporters in Fiji, Kiribati, and New Caledonia, and many processing methods assessed in this study also resulted in poorly salted products. Although processors use solid salt and aqueous salt as salting media, aqueous salt results in the decolored processed products, especially in *B. vitiensis* and *S. naso.* The erosion of the external body surface of sea cucumbers due to long-term contact with an aqueous (water) medium could be a possible reason for this observation.

Although many methods, i.e., smoke drying, freeze drying, microwave drying, and cabinet (electric) drying [14, 17, 22], are used to dry the processed sea cucumbers, sun drying is the most widely used method [6, 12, 17, 22]. Sri Lankan processors widely practice both sun drying and electric drying methods by considering the cost-effectiveness and practical applicability of these methods. Even though fast and convenient drying can be obtained by electric dryers, the sun-dried products of *H. spinifera* and *S. naso* were given high priority by exporters as such products are free from decoloration and unusual shrinkage. However, electric drying seems to be a good method for drying the processed *B. vitiensis*. Although sun drying is an easy and cost-effective method, there are many disadvantages, such as seasonality due to weather and difficulties in providing optimum drying conditions [22, 26]. Such problems can be easily eliminated in cabinet drying, but it is costly and causes some damage to the external appearance of the final products due to overshrinkages of individuals [27].

The proximate compositions of the processed *H. spinifera* samples were within a similar range to those reported by Nishanthan et al. [12]. As there was no published information on the proximate composition of *B. vitiensis* and *S. naso*, it is difficult to compare the present results. This study proved that processing methods have a significant influence on the proximate composition of *bêche-de-mer* and even a small deviation from the major processing steps is enough to change the proximate composition of the final products. Although exporters' prioritization of *bêche-de-mer* solely depends on their experience, their selection mostly confirms products with high nutritional value as well as the lowest moisture content.

In conclusion, Sri Lanka uses different combinations of processing steps to process fresh sea cucumbers into *bêche-de-mer*. The results of this study revealed that the product quality and nutritional composition of *bêche-de-mer* varied with respect to processing practices. Although different combinations of processing steps produce high-quality *bêche-de-mer* meeting the exporters' satisfaction, most of the practices yield poor-quality products. Therefore, the findings of this study will be useful to understand the effects of different processing practices on product quality and nutritional composition of *bêche-de-mer* as well as to select suitable processing methods to upgrade the product quality to meet the market demand.

## Figures and Tables

**Table 1:** Experiment design used to process three different sea cucumber species *H. spinifera*, *B. vitenensis*, and *S. naso*.

Steps	Species		
	*H. spinifera*	*B. vitiensis*	*S. naso*
Grading and cleaning	Grading and cleaning (*n* = 720)	Grading and cleaning (*n* = 360)	Grading and cleaning (*n* = 360)
Evisceration	Evisceration—making a small slit in the ventral body region (*n* = 720)	Evisceration—pressing the abdomen (*n* = 360)	Evisceration—carrying out after first boiling making a small slit in the anus (*n* = 360)
First boiling	First boiling (95°C) with two different boiling mediums (S—seawater and F—freshwater with added salt) and 3 different boiling times (T1—10 min, T2—15 min, and T3—20 min), respectively (1) Seawater (32 ppt) → 10 min, 15 min, and 20 min (*n* = 120) (2) Freshwater with added salt (32 ppt) → 10 min, 15 min, and 20 min (*n* = 120)	First boiling (95°C) with two different boiling mediums (S—seawater and F—freshwater with added salt) and 3 different boiling times (T1—10 min, T2—15 min, and T3—20 min), respectively (1) Seawater (32 ppt) → 10 min, 15 min, and 20 min (*n* = 60) (2) Freshwater with added salt (32 ppt) → 10 min, 15 min, and 20 min (*n* = 60)	First boiling (95°C) with two different boiling mediums (S—seawater and F—freshwater with added salt) and 3 different boiling times (T1—3 min, T2—6 min, and T3—9 min), respectively (1) Seawater (32 ppt) → 3 min, 6 min, and 9 min (*n* = 60) (2) Freshwater with added salt (32 ppt) → 3 min, 6 min, and 9 min (*n* = 60) Evisceration—carrying out after first boiling making a small slit in the anus (*n* = 360)
Removal of chalky materials	Removal of chalky materials using two different methods (D—soaking in freshwater and P—mixed with papaya powder) (1) Soaked in freshwater for 8 h and kept 2 h to drain the water, boiled 3 minutes, and brushed the chalky materials (*n* = 60) (2) Mixed papaya powder with boiled sea cucumbers (20 g per 100 individuals), kept 8-10 minutes to dissolve chalky materials (*n* = 60)	Not applicable	Not applicable
Salting	Salting with two different forms (S—solid salt and L—aqueous salt medium, respectively) (1) Salting with solid salt medium (*n* = 30) (2) Salting with aqueous salt medium (*n* = 30)	Salting with two different forms (S—solid salt and L—aqueous salt medium, respectively) (1) Salting with solid salt medium (*n* = 30) (2) Salting with aqueous salt medium (*n* = 30)	Salting with two different forms (S—solid salt and L—aqueous salt medium, respectively) (1) Salting with solid salt medium (*n* = 30) (2) Salting with aqueous salt medium (*n* = 30)
Second boiling	Second boiling for 10 minutes	Second boiling for 10 minutes	Second boiling for 5 minutes
Drying	Drying using two different methods (F—sun drying and T—use of electric dryer, respectively) (1) Sun drying for 5 days (*n* = 15) (2) Use of electric dryer for 15 h (*n* = 15)	Drying using two different methods (F—sun drying and T—use of electric dryer, respectively) (1) Sun drying for 5 days (*n* = 15) (2) Use of electric dryer for 15 h (*n* = 15)	Drying using two different methods (F—sun drying and T—use of electric dryer, respectively) (1) Sun drying for 3 days (*n* = 15) (2) Use of electric dryer for 12 h (*n* = 15)

**Table 2:** Initial weight, processed weight, percentage weight loss, parameters used to assess the quality of the processed products (percentages of damaged, % deformed, % visible patches, and % oversalted), and exporters' ranking for the high-quality *bêche-de-mer* of *H. spinifera*, *B. vitiensis*, and *S. naso*.

#	Species	Sample code	Sample size (*n*)	Initial weight (mean ± SD, g)	Processed weight (mean ± SD, g)	% Weight loss	% Damaged	% Deformed	% Visible patches	% Oversalted	Exporters' ranking
1	*H. spinifera*	F20PST	15	268.30 ± 46.37	25.3 ± 1.66	90.57	0.00	20.00	20.00	20.00	2
		F15DLT	15	253.06 ± 38.37	24.48 ± 2.36	90.33	0.00	20.00	0.00	56.33	5
		F10DLT	15	251.74 ± 55.68	23.78 ± 3.11	90.55	0.00	20.00	20.00	60.00	8
		F10DST	15	218.16 ± 47.88	30.78 ± 2.54	85.89	0.00	20.00	0.00	40.00	7
		F20PLF	15	245.50 ± 25.45	21.86 ± 3.59	91.10	0.00	16.67	16.67	33.33	3
		F10PLF	15	221.86 ± 13.59	21.16 ± 0.47	90.46	0.00	20.00	0.00	60.00	6
		S15DSF	15	226.84 ± 42.08	23.76 ± 4.07	89.53	0.00	20.00	0.00	40.00	4
		F20DSF	15	254.12 ± 45.52	23.4 ± 1.94	90.79	0.00	20.00	0.00	20.00	1
2	*B. vitiensis*	F10ST	15	272.56 ± 63.7	22.4 ± 3.67	91.78	0.00	16.67	0.00	50.00	2
		F15ST	15	299.2 ± 36.83	20.18 ± 0.48	93.26	0.00	0.00	20.00	40.00	1
		S15LT	15	288.6 ± 53.07	21.94 ± 4.43	92.40	0.00	20.00	40.00	20.00	5
		S10ST	15	255.2 ± 35.86	22.74 ± 5.68	91.09	0.00	0.00	0.00	20.00	3
		F10LT	15	257.4 ± 52.24	27.16 ± 8.23	89.45	0.00	20.00	40.00	40.00	6
		S20LF	15	223.8 ± 61.7	23.52 ± 3.35	89.49	0.00	0.00	20.00	20.00	3
		S15LF	15	288.6 ± 53.07	21.94 ± 4.43	92.40	0.00	20.00	20.00	40.00	4
		F20LF	15	275.4 ± 56.29	19.54 ± 2.66	92.90	0.00	0.00	40.00	20.00	7
3	*S. naso*	S6LF	15	48.6 ± 10.11	3.68 ± 0.98	92.43	0.00	20.00	0.00	0.00	6
		F6LT	15	47.0 ± 6.20	5.3 ± 1.36	88.72	0.00	16.67	16.67	0.00	5
		F3SF	15	49.0 ± 9.7	3.18 ± 0.43	93.51	0.00	0.00	0.00	0.00	2
		S6SF	15	46.8 ± 11.61	4.56 ± 1.17	90.26	0.00	0.00	14.29	14.29	3
		S3LF	15	47.2 ± 5.81	3.56 ± 0.96	92.46	0.00	16.67	16.67	33.33	7
		F6SF	15	47.6 ± 12.28	3.28 ± 0.44	93.11	0.00	0.00	0.00	0.00	4
		F9LF	15	49.4 ± 9.24	4.68 ± 0.79	90.53	0.00	16.67	16.67	0.00	4
		S3SF	15	47.4 ± 3.51	6.38 ± 0.83	86.54	0.00	0.00	0.00	0.00	1

Average weights are given in mean weight in grams ± standard deviation; the highest quality *bêche-de-mer* was given rank 1 in the exporters' ranking.

**Table 3:** Proximate composition of the differently processed export-quality *bêche-de-mer* of *H. spinifera*, *B. vitiensis*, and *S. naso.*

#	Species	Exporters' ranking	Sample code	% Moisture	% Protein (DM)	% Ash (DM)	% Fat (DM)
1	*H. spinifera*	1	F20DCF	18.92 ± 0.25^d^	55.55 ± 0.13^cd^	35.14 ± 0.5^ab^	1.4 ± 0.0^a^
		2	F20PST	19.94 ± 0.04^c^	58.68 ± 0.67^a^	33.04 ± 0.26^bc^	1.47 ± 0.02^a^
		3	F20PLF	20.46 ± 0.13^c^	56.78 ± 0.17^b^	32.52 ± 1.54^cd^	1.46 ± 0.02^a^
		4	S15DSF	17.9 ± 0.13^e^	58.47 ± 0.08^a^	31.23 ± 0.16^cd^	1.32 ± 0.04^b^
		5	F15DLT	18.32 ± 0.29^de^	55.82 ± 0.01^bcd^	35.91 ± 0.52^a^	1.26 ± 0.0^b^
		6	F10PLF	21.2 ± 0.13^b^	55.4 ± 0.2^d^	30.05 ± 0.38^d^	1.46 ± 0.01^a^
		7	F10DST	22.81 ± 0.26^a^	59.21 ± 0.07^a^	33.5 ± 0.14^abc^	1.3 ± 0.02^b^
		8	F10DLT	22.51 ± 0.05^a^	56.64 ± 0.33^bc^	31.58 ± 0.4^cd^	1.41 ± 0.0^a^
2	*B. vitiensis*	1	F15ST	19.78 ± 0.16^ab^	51.56 ± 0.21^a^	42.27 ± 0.7^a^	1.85 ± 0.01^cd^
		2	F10ST	20.14 ± 0.82^ab^	50.94 ± 0.42^a^	39.85 ± 0.03^b^	1.76 ± 0.05^d^
		3	S10ST	20.68 ± 0.1^a^	52.18 ± 0.7^a^	42.45 ± 0.42^a^	1.94 ± 0.02^bc^
		3	S20LF	19.08 ± 0.01^b^	52.45 ± 0.29^a^	41.27 ± 0.7^ab^	1.91 ± 0.01^c^
		4	S15LF	19.97 ± 0.06^ab^	49.97 ± 1.98^a^	42.77 ± 0.09^a^	1.78 ± 0.03^d^
		5	S15LT	19.66 ± 0.21^ab^	52.56 ± 1^a^	41.83 ± 0.18^a^	2.03 ± 0.01^ab^
		6	F10LT	21.06 ± 0.49^a^	52.33 ± 0.67^a^	41.68 ± 0.06^a^	1.87 ± 0.01^c^
		7	F20LF	19.26 ± 0.06^b^	50.16 ± 0.23^a^	41.99 ± 0.01^a^	2.04 ± 0.02^a^
3	*S. naso*	1	S3SF	14.67 ± 0.62^b^	51.90 ± 0.44^a^	44.22 ± 0.49^ab^	2.65 ± 0.15^a^
		2	F3SF	17.89 ± 1.01^a^	48.93 ± 0.1^de^	42.26 ± 0.76^c^	2.29 ± 0.02^c^
		3	S6SF	16.36 ± 0.23^ab^	50.71 ± 0.22^bc^	42.22 ± 0.11^c^	2.59 ± 0.01^ab^
		4	F6SF	17.41 ± 0.25^ab^	48.1 ± 0.07^e^	42.14 ± 0.09^c^	2.61 ± 0.08^ab^
		4	F9LF	16.57 ± 0.51^ab^	50.1 ± 0.44^cd^	44.62 ± 0.44^a^	2.37 ± 0.06^bc^
		5	F6LT	16.56 ± 0.68^ab^	48.89 ± 0.26^de^	43.21 ± 0.67^abc^	2.46 ± 0.02^abc^
		6	S6LF	15.17 ± 0.81^ab^	51.84 ± 0.47^ab^	42.46 ± 0.06^c^	2.38 ± 0.02^bc^
		7	S3LF	15.02 ± 0.99^b^	48.95 ± 0.14^de^	42.72 ± 0.01^bc^	2.38 ± 0.02^bc^

^∗∗^Values in the same column for each species bearing different letters are significantly different (*p* < 0.05). The proximate results are expressed in % (m) dry weight basis.

## Data Availability

The data used to support the findings of this study are included within the article.

## Supplementary Materials

Supplementary Materials Table 1: initial weight (mean ± SD), processed weight (mean ± SD), weight loss (%), and percentages of damaged, deformed, patched, and oversalted individuals of *H. spinifera* processed using different processing methods (*n* = 48) and selection and ranking of export-quality *bêche-de-mer* as per exporters' (*n* = 9) choice. Table 2: initial weight (mean ± SD), processed weight (mean ± SD), weight loss (%), and percentages of damaged, deformed, patched, and oversalted individuals of *B. vitiensis* processed using different processing methods (*n* = 24) and selection and ranking of export-quality *bêche-de-mer* as per exporters' (*n* = 9) choice. Table 3: initial weight (mean ± SD), processed weight (mean ± SD), weight loss (%), and percentages of damaged, deformed, patched, and oversalted individuals of *S. naso* processed using different processing methods (*n* = 24) and selection and ranking of export-quality *bêche-de-mer* as per exporters' (*n* = 9) choice.
