# Supplementary material for: Impact of Different Combinations of Processing Steps on Product Quality and Proximate Composition of Bêche-de-mer: A Case Study from Sri Lanka
Source: Int J Food Sci. 2022 Aug 9;2022:7877050. doi: 10.1155/2022/7877050 (PMC9420059; doi:10.1155/2022/7877050)
Supplement: Supplementary Materials — Table 1: initial weight (mean ± SD), processed weight (mean ± SD), weight loss (%), and percentages of damaged, deformed, patched, and oversalted individuals of H. spinifera processed using different processing methods (n = 48) and selection and ranking of export-quality bêche-de-mer as per exporters' (n = 9) choice. Table 2: initial weight (mean ± SD), processed weight (mean ± SD), weight loss (%), and percentages of damaged, deformed, patched, and oversalted individuals of B. vitiensis processed using different processing methods (n = 24) and selection and ranking of export-quality bêche-de-mer as per exporters' (n = 9) choice. Table 3: initial weight (mean ± SD), processed weight (mean ± SD), weight loss (%), and percentages of damaged, deformed, patched, and oversalted individuals of S. naso processed using different processing methods (n = 24) and selection and ranking of export-quality bêche-de-mer as per exporters' (n = 9) choice. [file 7877050.f1.docx]

Supplementary table 1: Initial weight (mean ± SD) , processed weight (mean ± SD), weight loss (%) and percentages of damaged, deformed, patched and over salted individuals of *H. spinifera* processed using different processing methods (n=48) and selection and ranking of export quality bêche-de-mer as per exporters (n=9) choice.

| **#** | **Sample**  **Code** | **Initial**  **Weight (g)** | **Processed weight**  **(g)** | **% weight loss** | **% Damaged** | **% deformed** | **% visible patches** | **% over salted** | **Exporters selection and ranking** |
| --- | --- | --- | --- | --- | --- | --- | --- | --- | --- |
| 1 | **F20PST** | **268.3±46.37** | **25.3±1.66** | **90.57** | **0.00** | **20.00** | **20.00** | **20.00** | **✓ (2)** |
| 2 | F20PLT | 245.15±38.45 | 18.73±2.36 | 92.36 | 0.00 | 33.33 | 50.00 | 16.67 | 🗶 |
| 3 | F10PST | 235.5±74.88 | 21.43±1.12 | 90.90 | 40.00 | 40.00 | 60.00 | 40.00 | 🗶 |
| 4 | F10PLT | 256.23±13.8 | 16.7±2.07 | 93.48^a^ | 0.00 | 50.00 | 50.00 | 50.00 | 🗶 |
| 5 | F15PST | 260.86±35.52 | 22.94±1.65 | 91.21 | 20.00 | 40.00 | 40.00 | 40.00 | 🗶 |
| 6 | F15PLT | 252.76±17.98 | 15.08±1.22 | 94.03 | 20.00 | 60.00 | 80.00 | 40.00 | 🗶 |
| 7 | S15PST | 251.78±35.89 | 15.78±2.12 | 93.73 | 0.00 | 40.00 | 60.00 | 0.00 | 🗶 |
| 8 | S20PLT | 252.78±17.92 | 21.96±2.11 | 91.31 | 0.00 | 20.00 | 40.00 | 60.00 | 🗶 |
| 9 | S20PST | 233.03±43.09 | 22.2±2.72 | 90.47 | 50.00 | 66.67 | 66.67 | 16.67 | 🗶 |
| 10 | S15PLT | 256.6±55.86 | 20.4±3.57 | 92.05 | 25.00 | 25.00 | 25.00 | 50.00 | 🗶 |
| 11 | S10PLT | 246.55±42.22 | 22.93±4.02 | 90.70 | 0.00 | 25.00 | 75.00 | 50.00 | 🗶 |
| 12 | S10PST | 254.58±12.59 | 22.9±3.8 | 91.00 | 0.00 | 20.00 | 40.00 | 40.00 | 🗶 |
| 13 | S10DLT | 272.2±70.0 | 26.37±1.1 | 90.31 | 50.00 | 33.33 | 16.67 | 50.00 | 🗶 |
| 14 | S10DST | 210.13±46.97 | 17.33±2.92 | 91.75 | 20.00 | 40.00 | 60.00 | 60.00 | 🗶 |
| 15 | F15DST | 248.24±43.0 | 19.46±0.95 | 92.16 | 20.00 | 40.00 | 60.00 | 60.00 | 🗶 |
| 16 | **F15DLT** | **253.06±38.37** | **24.48±2.36** | **90.33** | **0.00** | **20.00** | **0.00** | **50.00** | **✓ (5)** |
| 17 | S15DST | 233.4±56.81 | 22.8±1.24 | 90.23 | 16.67 | 66.67 | 16.67 | 50.00 | 🗶 |
| 18 | S15DLT | 235.96±35.46 | 20.88±4.05 | 91.15 | 40.00 | 60.00 | 40.00 | 80.00 | 🗶 |
| 19 | **F10DLT** | **251.74±55.68** | **23.78±3.11** | **90.55** | **0.00** | **20.00** | **20.00** | **60.00** | **✓ (8)** |
| 20 | **F10DST** | **218.16±47.88** | **30.78±2.54** | **85.89** | **0.00** | **20.00** | **0.00** | **60.00** | **✓ (7)** |
| 21 | F20DST | 244.22±53.0 | 24.3±2.05 | 90.05 | 14.29 | 71.43 | 57.14 | 57.14 | 🗶 |
| 22 | F20DLT | 224.82±46.8 | 16.98±1.59 | 92.45 | 16.67 | 33.33 | 66.67 | 66.67 | 🗶 |
| 23 | S20DLT | 228.68±37.59 | 15.94±2.13 | 93.03 | 20.00 | 40.00 | 40.00 | 60.00 | 🗶 |
| 24 | S20DST | 237.98±7.36 | 14.03±2.64 | 94.11 | 20.00 | 60.00 | 100.00 | 20.00 | 🗶 |
| 25 | F20PSF | 246.88±21.11 | 20.85±2.09 | 91.55 | 33.33 | 66.67 | 0.00 | 50.00 | 🗶 |
| 26 | **F20PLF** | **245.5±25.45** | **21.86±3.59** | **91.10** | **0.00** | **16.67** | **16.67** | **33.33** | **✓ (3)** |
| 27 | F10PSF | 237.68±25.06 | 17.63±2.09 | 92.58 | 20.00 | 40.00 | 0.00 | 40.00 | 🗶 |
| 28 | **F10PLF** | **221.86±13.59** | **21.16±0.47** | **90.46** | **0.00** | **20.00** | **0.00** | **60.00** | **✓(6)** |
| 29 | F15PSF | 212.83±53.02 | 21.55±2.15 | 89.87 | 20.00 | 40.00 | 80.00 | 40.00 | 🗶 |
| 30 | F15PLF | 247.33±26.09 | 22.5±2.41 | 90.90 | 40.00 | 40.00 | 60.00 | 40.00 | 🗶 |
| 31 | S15PSF | 228.34±23.58 | 15.82±2.04 | 93.07 | 20.00 | 60.00 | 40.00 | 20.00 | 🗶 |
| 32 | S20PLF | 216.1±48.8 | 19.78±1.28 | 90.85 | 0.00 | 20.00 | 40.00 | 40.00 | 🗶 |
| 33 | S20PSF | 227.5±47.12 | 19.58±1.01 | 91.40 | 0.00 | 60.00 | 60.00 | 60.00 | 🗶 |
| 34 | S15PLF | 273.2±41.55 | 24.88±6.27 | 90.89 | 20.00 | 20.00 | 40.00 | 60.00 | 🗶 |
| 35 | S10PSF | 265.74±29.38 | 22.72±4.15 | 91.45 | 20.00 | 40.00 | 60.00 | 20.00 | 🗶 |
| 36 | S10PLF | 224.0±34.7 | 27.02±1.68 | 87.94 | 0.00 | 20.00 | 60.00 | 40.00 | 🗶 |
| 37 | F10DSF | 259.53±54.93 | 22.75±1.51 | 91.23^ab^ | 0.00 | 20.00 | 20.00 | 100.00 | 🗶 |
| 38 | F10DLF | 242.98±46.17 | 23.13±1.76 | 90.48^abc^ | 0.00 | 20.00 | 60.00 | 60.00 | 🗶 |
| 39 | S15DLF | 239.15±26.3 | 24.55±2.37 | 89.73 | 20.00 | 60.00 | 80.00 | 60.00 | 🗶 |
| 40 | **S15DSF** | **226.84±42.08** | **23.76±4.07** | **89.53** | **0.00** | **20.00** | **0.00** | **40.00** | **✓ (4)** |
| 41 | F15DLF | 235.15±22.27 | 21.4±6.53 | 90.90 | 0.00 | 25.00 | 25.00 | 50.00 | 🗶 |
| 42 | F15DSF | 223.96±38.07 | 27.48±3.69 | 87.73 | 0.00 | 80.00 | 40.00 | 60.00 | 🗶 |
| 43 | S10DSF | 220.5±30.19 | 22.28±2.59 | 89.90 | 20.00 | 40.00 | 80.00 | 60.00 | 🗶 |
| 44 | S10DLF | 269.93±6.54 | 22.95±2.25 | 91.50 | 0.00 | 20.00 | 80.00 | 60.00 | 🗶 |
| 45 | **F20DSF** | **254.12±45.52** | **23.4±1.94** | **90.79** | **0.00** | **20.00** | **0.00** | **20.00** | **✓ (1)** |
| 46 | F20DLF | 262.48±35.84 | 15.78±2.12 | 93.99 | 20.00 | 60.00 | 80.00 | 40.00 | 🗶 |
| 47 | S20DLF | 218.33±10.77 | 15.63±1.21 | 92.84 | 20.00 | 20.00 | 60.00 | 60.00 | 🗶 |
| 48 | S20DSF | 225.78±20.16 | 21.96±4.33 | 90.27 | 0.00 | 40.00 | 60.00 | 80.00 | 🗶 |

** ✓ = sample selected by exporters, 🗶 = sample rejected by exporters

Supplementary table 2: Initial weight (mean ± SD) , processed weight (mean ± SD), weight loss (%) and percentages of damaged, deformed, patched and over salted individuals of *B. vitiensis* processed using different processing methods (n=24) and selection and ranking of export quality bêche-de-mer as per exporters (n=9) choice.

| **#** | **Sample**  **Code** | **Initial**  **Weight (g)** | **Processed weight (g)** | **% weight loss** | **% Damaged** | **% deformed** | **% visible patches** | **% over salted** | **Exporters selection and ranking** |
| --- | --- | --- | --- | --- | --- | --- | --- | --- | --- |
| 1 | **F10ST** | **272.56±63.7** | **22.4±3.67** | **91.78** | **0.00** | **16.67** | **0.00** | **50.00** | **✓ (2)** |
| 2 | F20LT | 258.4±26.39 | 21.04±6.49 | 91.86 | 0.00 | 20.00 | 60.00 | 60.00 | 🗶 |
| 3 | S20LT | 275.8±43.69 | 20.72±3.27 | 92.49 | 0.00 | 60.00 | 0.00 | 60.00 | 🗶 |
| 4 | **F15ST** | **299.2±36.83** | **20.18±0.48** | **93.26** | **0.00** | **0.00** | **20.00** | **40.00** | **✓ (1)** |
| 5 | S10LT | 219.2±28.93 | 20.92±3.58 | 90.46 | 0.00 | 80.00 | 20.00 | 60.00 | 🗶 |
| 6 | D15ST | 266.0±60.57 | 16.8±1.68 | 93.68 | 0.00 | 60.00 | 40.00 | 60.00 | 🗶 |
| 7 | **S15LT** | **248.2±32.6** | **26.38±2.43** | **89.37** | **0.00** | **20.00** | **20.00** | **40.00** | **✓ (5)** |
| 8 | F15LT | 276.6±53.86 | 32.16±7.08 | 88.37 | 0.00 | 20.00 | 60.00 | 60.00 | 🗶 |
| 9 | **S10ST** | **255.2±35.86** | **22.74±5.68** | **91.09** | **0.00** | **0.00** | **0.00** | **20.00** | **✓ (3)** |
| 10 | S20ST | 213.6±11.72 | 22.36±3.64 | 89.53 | 0.00 | 62.50 | 25.00 | 62.50 | 🗶 |
| 11 | **F10LT** | **257.4±52.24** | **27.16±8.23** | **89.45** | **0.00** | **20.00** | **40.00** | **40.00** | **✓ (6)** |
| 12 | F20ST | 242.8±50.5 | 20.8±5.78 | 91.43 | 0.00 | 85.71 | 0.00 | 71.43 | 🗶 |
| 13 | S15SF | 252.0±46.81 | 16.3±2.67 | 93.53 | 0.00 | 80.00 | 20.00 | 20.00 | 🗶 |
| 14 | F10LF | 276.2±30.65 | 19.1±3.57 | 93.08 | 20.00 | 40.00 | 20.00 | 40.00 | 🗶 |
| 15 | F15SF | 249.0±51.94 | 25.74±4.13 | 89.66 | 20.00 | 60.00 | 60.00 | 60.00 | 🗶 |
| 16 | F15LF | 288.2±26.13 | 23.2±5.16 | 91.95 | 0.00 | 20.00 | 60.00 | 80.00 | 🗶 |
| 17 | S10SF | 264.0±32.56 | 20.16±5.74 | 92.36 | 0.00 | 40.00 | 40.00 | 40.00 | 🗶 |
| 18 | F10SF | 236.4±12.9 | 21.0±4.27 | 91.12 | 0.00 | 60.00 | 0.00 | 40.00 | 🗶 |
| 19 | S20SF | 259.6±18.98 | 13.94±2.81 | 94.63 | 0.00 | 77.78 | 11.11 | 22.22 | 🗶 |
| 20 | F20SF | 271.8±48.05 | 24.94±3.27 | 90.82 | 0.00 | 60.00 | 40.00 | 40.00 | 🗶 |
| 21 | S10LF | 243.2±54.87 | 19.04±6.13 | 92.17 | 0.00 | 40.00 | 40.00 | 60.00 | 🗶 |
| 22 | **S20LF** | **223.8±61.7** | **23.52±3.35** | **89.49** | **0.00** | **0.00** | **20.00** | **20.00** | **✓ (3)** |
| 23 | **S15LF** | **288.6±53.07** | **21.94±4.43** | **92.40** | **0.00** | **20.00** | **40.00** | **20.00** | **✓ (4)** |
| 24 | **F20LF** | **275.4±56.29** | **19.54±2.66** | **92.90** | **0.00** | **0.00** | **40.00** | **20.00** | **✓ (7)** |

** ✓ = sample selected by exporters, 🗶 = sample rejected by exporters

Supplementary table 3: Initial weight (mean ± SD) , processed weight (mean ± SD), weight loss (%) and percentages of damaged, deformed, patched and over salted individuals of *S. naso* processed using different processing methods (n=24) and selection and ranking of export quality bêche-de-mer as per exporters (n=9) choice.

| **#** | **Sample**  **Code** | **Initial**  **Weight (g)** | **Processed weight**  **(g)** | **% weight loss** | **% Damaged** | **% deformed** | **% visible patches** | **% over salted** | **Exporters selection and ranking** |
| --- | --- | --- | --- | --- | --- | --- | --- | --- | --- |
| 1 | **S6LF** | **48.6±10.11** | **3.68±0.98** | **92.43** | **0.00** | **20.00** | **0.00** | **0.00** | **✓ (6)** |
| 2 | F3ST | 45.6±10.24 | 6.5±2.42 | 85.75 | 0.00 | 50.00 | 66.67 | 0.00 | 🗶 |
| 3 | F6ST | 43.4±7.23 | 2.38±0.19 | 94.52 | 0.00 | 33.33 | 50.00 | 0.00 | 🗶 |
| 4 | S3LT | 44.4±6.19 | 3.32±0.44 | 92.52 | 0.00 | 50.00 | 16.67 | 0.00 | 🗶 |
| 5 | S6ST | 47.0±6.2 | 3.06±0.41 | 93.49 | 0.00 | 42.86 | 57.14 | 0.00 | 🗶 |
| 6 | F9LT | 46.2±6.91 | 3.08±0.54 | 93.33 | 14.29 | 28.57 | 42.86 | 0.00 | 🗶 |
| 7 | F9ST | 44.6±6.19 | 2.88±0.5 | 93.54 | 7.69 | 46.15 | 15.38 | 0.00 | 🗶 |
| 8 | **F6LT** | **47.0±6.2** | **5.3±1.36** | **88.72** | **0.00** | **16.67** | **16.67** | **0.00** | **✓ (5)** |
| 9 | S9ST | 48.0±9.22 | 2±0.39 | 95.83 | 6.67 | 46.67 | 53.33 | 0.00 | 🗶 |
| 10 | S9LT | 46.2±5.45 | 1.82±0.13 | 96.06 | 0.00 | 60.00 | 40.00 | 0.00 | 🗶 |
| 11 | F3LT | 45.2±8.44 | 3.68±0.8 | 91.86 | 25.00 | 62.50 | 25.00 | 0.00 | 🗶 |
| 12 | S3ST | 42.0±7.71 | 3.48±0.36 | 91.71 | 0.00 | 33.33 | 16.67 | 0.00 | 🗶 |
| 13 | F9SF | 42.0±2.83 | 2.34±0.18 | 94.43 | 6.67 | 53.33 | 33.33 | 0.00 | 🗶 |
| 14 | **F3SF** | **49.0±9.7** | **3.18±0.43** | **93.51** | **0.00** | **0.00** | **0.00** | **0.00** | **✓ (2)** |
| 15 | S9LF | 48.4±11.61 | 2.22±0.31 | 95.41 | 15.38 | 53.85 | 38.46 | 0.00 | 🗶 |
| 16 | **S6SF** | **46.8±11.61** | **4.56±1.17** | **90.26** | **0.00** | **0.00** | **14.29** | **14.29** | **✓ (3)** |
| 17 | S9SF | 42.0±14.18 | 2.62±0.61 | 93.76 | 0.00 | 55.56 | 22.22 | 0.00 | 🗶 |
| 18 | F6LF | 46.6±5.18 | 3.58±0.86 | 92.32 | 0.00 | 20.00 | 40.00 | 0.00 | 🗶 |
| 19 | F3LF | 47.0±2.83 | 3.76±0.18 | 92.00 | 20.00 | 20.00 | 80.00 | 0.00 | 🗶 |
| 20 | S6LF | 42.8±2.17 | 3.32±0.43 | 92.24 | 0.00 | 33.33 | 50.00 | 0.00 | 🗶 |
| 21 | **S3LF** | **47.2±5.81** | **3.56±0.96** | **92.46** | **0.00** | **16.67** | **16.67** | **33.33** | **✓ (7)** |
| 22 | **F6SF** | **47.6±12.28** | **3.28±0.44** | **93.11** | **0.00** | **0.00** | **20.00** | **0.00** | **✓ (4)** |
| 23 | **F9LF** | **49.4±9.24** | **4.68±0.79** | **90.53** | **0.00** | **16.67** | **16.67** | **0.00** | **✓ (4)** |
| 24 | **S3SF** | **47.4±3.51** | **6.38±0.83** | **86.54** | **0.00** | **0.00** | **0.00** | **0.00** | **✓ (1)** |

** ✓ = sample selected by exporters, 🗶 = sample rejected by exporters
